# Short-Term Atorvastatin Therapy in Healthy Individuals Results in Unaltered Plasma MMP Levels and Disrupted MMP-7 Correlation with Blood Lipids and Blood Count-Derived Inflammatory Markers

**DOI:** 10.3390/jcm13164743

**Published:** 2024-08-13

**Authors:** Ion Bogdan Mănescu, Măriuca Mănescu, Laura Iulia Bărcuțean, Liliana Demian, Minodora Dobreanu

**Affiliations:** 1 Department of Laboratory Medicine, George Emil Palade University of Medicine, Pharmacy, Science, and Technology of Targu Mures, 38 Gheorghe Marinescu, 540142 Targu Mures, Romania; bogdan.manescu@umfst.ro (I.B.M.); minodora.dobreanu@umfst.ro (M.D.); 2 Department of Pediatrics, Emergency County Clinical Hospital of Targu Mures, 50 Gheorghe Marinescu, 540136 Targu Mures, Romania; 3 Department of Neurology, George Emil Palade University of Medicine, Pharmacy, Science, and Technology of Targu Mures, 38 Gheorghe Marinescu, 540142 Targu Mures, Romania; laurabarcutean@gmail.com; 4 Neurology 1 Clinic, Emergency County Clinical Hospital of Targu Mures, 50 Gheorghe Marinescu, 540136 Targu Mures, Romania; 5 Clinical Laboratory, Emergency County Clinical Hospital of Targu Mures, 50 Gheorghe Marinescu, 540136 Targu Mures, Romania; lilidemian@yahoo.com; 6 Immunology Laboratory, Center for Advanced Medical and Pharmaceutical Research (CCAMF), George Emil Palade University of Medicine, Pharmacy, Science, and Technology of Targu Mures, 38 Gheorghe Marinescu, 540142 Targu Mures, Romania

**Keywords:** atherosclerosis, atorvastatin, healthy individual, inflammation, lipids, matrix metalloproteinase, MMP-2, MMP-7, MMP-9

## Abstract

**Background**: Matrix metalloproteinases (MMPs) play an important role in the pathophysiology of atherosclerosis. Reportedly, statins can decrease MMP activity in patients with atherosclerotic cardiovascular disease, but this effect has not been studied in healthy individuals. **Methods**: MMPs 2, 7, and 9 and several other parameters were measured before and after a four-week course of moderate-dose atorvastatin (20 mg/day) in 21 healthy individuals. **Results**: Atorvastatin treatment resulted in lower total cholesterol, LDL-cholesterol, non-HDL-cholesterol, and triglycerides (*p* < 0.001 for all), but higher levels of plasma enzymes AST, ALT, CK, and LDH (*p* < 0.05 for all). No effect of atorvastatin on plasma MMP median concentrations was recorded. Before treatment, moderate positive significant correlations were found between MMP-7 and age, blood lipids, and blood count-derived inflammatory markers. Pre-treatment MMP-7 was best predicted by the total cholesterol-to-HDL cholesterol ratio in a remnant cholesterol-weighted least squares regression model. After atorvastatin treatment, MMP-7 no longer correlated with these markers. **Conclusions**: While the effect of statins on plasma MMPs in atherosclerosis is controversial, short-term moderate-dose atorvastatin treatment does not seem to affect levels of MMPs 2, 7, and 9 in healthy individuals. However, an intriguing correlation between MMP-7 and atherosclerosis-related blood lipids and neutrophil-associated inflammatory biomarkers seems to be disrupted by atorvastatin independently of hsCRP, possibly via pleiotropic effects.

## 1. Introduction

Cardiovascular diseases (CVDs) remain the primary cause of mortality globally [1]. Among CVDs, ischemic heart diseases, including myocardial infarction (MI), are responsible for the majority of deaths [1]. MI is often preceded by atherosclerosis in the coronary arteries, a gradual process of plaque formation that typically unfolds over long periods of time, characterized by a “preclinical” phase with no noticeable symptoms. The rupture of coronary atherosclerotic plaques can result in coronary thrombosis, leading to type 1 MI. Many studies have shown that the development and stability of atherosclerotic plaques are dependent on the architecture and integrity of their extracellular matrix (ECM), which comprises proteins like elastin and collagen [2]. Matrix metalloproteinases (MMPs), a class of enzymes that can cleave ECM proteins, play a pivotal role in the remodeling process associated with atherosclerosis and MI [2,3]. The degradation of ECM proteins by MMPs can promote atherogenesis and subsequently destabilize the fibrous cap of already formed atherosclerotic plaques [2,3,4]. Moreover, primarily through the degradation of ECM proteins, MMPs are involved in various other biological processes that have been linked to atherosclerosis, such as angiogenesis, immune response regulation, endothelial dysfunction, vascular inflammation, and cell activation, differentiation, and migration [2,3,4].

Given their importance in plaque development and stability, MMPs have undergone extensive investigation in the context of atherosclerosis. A plethora of in vitro and animal model studies have shown statins indeed reduce the expression of key MMPs and attenuate their production and release from various cell types, including macrophages and vascular smooth muscle cells [2,3,4,5]. Consequently, it is now widely accepted that statins effectively modulate MMP activity at the local level, contributing to plaque stabilization [2,3]. However, at the systemic cardiovascular level, it remains unclear whether statin therapy leads to reductions in circulating MMP levels and whether such reductions are associated with diminished cardiovascular risk. Further complicating the interpretation of these findings is the variation in study populations and methodologies, as well as the lack of data from healthy cohorts for reference. In this brief report, we aimed to address this gap by investigating the impact of statins on plasma MMP levels in healthy individuals. We consider this information relevant for interpreting subsequent findings from more extensive studies involving patients with cardiovascular diseases.

## 2. Materials and Methods

### 2.1. Study Design and Sample Collection

This study involved 21 clinically healthy individuals aged 25–63, who gave written informed consent for participation. Exclusion criteria: below 18 years of age, any acute or chronic inflammatory condition, any pathology known to interfere with lipid metabolism (e.g., diseases of the liver, kidney, thyroid, etc.), and any current medication (especially lipid-lowering drugs). After an overnight fast, peripheral blood samples were collected in sodium heparin tubes (BD Vacutainer^®^, cat. no. 367884; Eysins, Switzerland). The samples were stored and transported according to local laboratory protocols and processed within four hours from collection. First, a complete blood count (CBC) was performed for each participant on a SYSMEX XS-800i (Norderstedt, Germany) automated hematology analyzer. Samples were then centrifuged for 10 min (3000 RPM, room temperature) and plasma was separated and stored until analysis at −80 °C in 2.0 mL cryo-tubes (Ratiolab, cat. no. 5110122; Dreieich, Germany).

### 2.2. Statin Treatment

We selected atorvastatin from among the available statins for several key reasons. First, atorvastatin is highly prevalent among the population from which we intend to recruit cardiovascular patients for future studies. According to unpublished data from our research group, among patients with known coronary heart disease who are on statin therapy, 55% use atorvastatin, 38% use rosuvastatin, and 7% use simvastatin. Second, given our interest in the pleiotropic effects of statins and our group’s involvement in in vitro cellular experiments, we anticipated that atorvastatin would exhibit enhanced cellular penetration due to its lipophilic nature, in contrast to rosuvastatin, which is hydrophilic.

Participants were not prescribed atorvastatin per se; however, after signing the informed consent, they were supplied with a box containing 30 tablets of atorvastatin 20 mg. This moderate dosage was considered both medically and ethically acceptable. Participants were instructed to administer one tablet daily. Detailed information regarding the safety and potential side effects of statins was provided to each participant. They were instructed not to double the dosage if they missed a day and to discontinue the medication immediately and notify the study organizer in case of any serious side effects (defined as anything more serious than mild myalgia). The timing of medication self-administration was left to the discretion of each participant (e.g., morning/before sleep). Throughout the study, the organizers conducted regular welfare checks to monitor side effects and provided reminders to enhance participant compliance. At the end of the 28-day period, fasting blood samples were collected, CBCs were performed again, and plasma was separated and stored according to the previously described protocol.

### 2.3. MMP Multiplex Assay

For the assessment of MMP levels, plasma samples previously stored at −80 °C were initially thawed and vigorously vortexed. Samples were then processed according to the manufacturer’s instructions, using a commercially available multiplex bead-based immunoassay kit (MILLIPLEX^®^ Human MMP Magnetic Bead Panel 2; Merck-Millipore cat. no. HMMP2MAG-55K; Burlington, MA, USA). Processed samples were analyzed on a FLEXMAP 3D^®^ analyzer (Luminex^®^ xMAP^®^ technology; Luminex Corporation, Austin, TX, USA) and data acquisition was performed using xPONENT^®^ software version 4.3. In this study, three MMP types were analyzed: MMP-2, MMP-7, and MMP-9. All results were converted from the original unit of measurement (pg/mL) and are reported in ng/mL.

### 2.4. Lipid Profile and Chemistry Assays

For the assessment of the standard lipid profile and other chemistry parameters, plasma samples previously stored at −80 °C were initially thawed and vigorously vortexed. Using a Cobas Integra 400 Plus analyzer (Roche, Basel, Switzerland), the standard lipid profile was tested for all samples: total cholesterol (TC), low-density lipoprotein-cholesterol (dLDLC, measured directly), high-density lipoprotein-cholesterol (HDLC), non-HDLC (calculated: TC − HDLC), and triglycerides (TG). Additionally, the following analytes were measured on the same instrument: albumin, alanine transaminase (ALT), amylase, aspartate aminotransferase (AST), creatinine, creatine kinase (CK), gamma-glutamyl transferase (GGT), glucose, and lactate dehydrogenase (LDH). Lipoprotein(a) was measured by nephelometry on a BN ProSpec analyzer (Siemens, Munich, Germany). High-sensitivity C-reactive protein (hsCRP) was measured on an Alinity ci analyzer (Abbott, Chicago, IL, USA).

### 2.5. Statistical Analysis

Statistical distribution of datasets was tested using the Kolmogorov–Smirnov test for normality. The concentrations of analytes before and after treatment were compared using either the paired samples *t*-test or Wilcoxon test, selected based on the distribution of datasets. Additional derived parameters including the De Ritis ratio (AST/ALT), TC/HDLC, LDLC/HDLC, atherogenic index of plasma [AIP = log_10_(TG/HDLC)], monocyte-to-lymphocyte ratio (MLR), neutrophil-to-lymphocyte ratio (NLR), derived NRL [dNLR = (Neutrophil count)/(WBC − Neutrophil count)], systemic inflammation response index [SIRI = (monocyte count × neutrophil count)/lymphocyte count], neutrophil-to-HDLC ratio (NHR), monocyte-to-HDLC ratio (MHR), and lymphocyte-to-HDLC ratio (LHR) were also compared.

Correlation tests were conducted between datasets at each time point: pre- and post-treatment. Additionally, changes in each parameter were calculated using the formula Δ% = 100 × (post-treatment concentration − pre-treatment concentration)/pre-treatment concentration, and correlations were assessed between datasets of these differences. Correlation results are reported as correlation coefficients, either Pearson’s r or Spearman’s ρ, depending on the characteristics of each dataset.

All statistical testing was performed using MedCalc^®^ Statistical Software version 20.104 (MedCalc Software Ltd., Ostend, Belgium). Statistical significance was set at *p* ≤ 0.05 and *p*-values between 0.05 and 0.10 were considered marginally significant and are also reported. Significance thresholds are represented as follows: ^#^ *p* ≤ 0.10, * *p* ≤ 0.05, ** *p* ≤ 0.01, *** *p* ≤ 0.005.

### 2.6. Logistics and Ethics Approval

Blood samples were collected at the Emergency County Clinical Hospital of Targu Mures (Romania). Sample processing, storage, and analysis were performed at the Center for Advanced Medical and Pharmaceutical Research (CCAMF) of the George Emil Palade University of Medicine, Pharmacy, Science, and Technology of Targu Mures (Romania). The study was approved by the university’s Research Ethics Committee (approval no. 1849/15.09.2022).

## 3. Results

General data and laboratory parameters at baseline and after statin treatment are presented in Table 1. Notably, without their prior knowledge, almost half of the participants had dLDLC levels above 130 mg/dL before treatment; however, none of the participants had dLDLC levels above 190 mg/dL. Also, about 75% of participants had Lp(a) levels below the limit of quantification.

Correlations of MMP levels with lipid and blood count parameters before and after statin treatment are detailed in Table 2. Significant simple linear regression models for the levels of MMP-7 before treatment are provided in Table 3. No significant multiple regression model was found to surpass the simple regression model based on TC/HDLC in predicting plasma levels of MMP-7 before treatment. However, the performance of the TC/HDLC simple linear regression model was significantly improved by adding various variables as weights: WBC (R^2^ = 0.44, *p* = 0.0011); dNLR and NLR (both R^2^ = 0.46, *p* = 0.0007); NHR and SIRI (both R^2^ = 0.47, *p* = 0.0006); age, neutrophil count, and triglyceride (all R^2^ = 0.48, *p* = 0.0005); and remnant cholesterol (R^2^ = 0.53, *p* = 0.0002). The remnant cholesterol-weighted least squares regression model for MMP-7 based on TC/HDLC was: Y = 2.01 + 3.88X. After statin treatment, no significant regression models were identified for MMP-7.

Before treatment, high-sensitivity C-reactive protein (hsCRP) was also weakly/moderately correlated with blood lipids, but not with neutrophil-related parameters: TC (ρ = 0.25, *p* = 0.27), dLDLC (ρ = 0.34, *p* = 0.14), HDLC (ρ = −0.38, *p* = 0.09), non-HDLC (ρ = 0.42, *p* = 0.06), remnant cholesterol (ρ = 0.48, *p* = 0.03), TG (ρ = 0.41, *p* = 0.07), AIP (ρ = 0.48, *p* = 0.03), TC/HDLC (ρ = 0.57, *p* = 0.008), and dLDLC/HDLC (ρ = 0.54, *p* = 0.01). Contrarily to MMP-7, hsCRP maintained and even improved its correlations with blood lipids after treatment, except for HDLC: TC (ρ = 0.30, *p* = 0.19), dLDLC (ρ = 0.47, *p* = 0.03), HDLC (ρ = 0.00, *p* = 0.99), non-HDLC (ρ = 0.49, *p* = 0.02), remnant cholesterol (ρ = 0.46, *p* = 0.03), TG (ρ = 0.44, *p* = 0.04), AIP (ρ = 0.44, *p* = 0.05), TC/HDLC (ρ = 0.57, *p* = 0.008), and dLDLC/HDLC (ρ = 0.54, *p* = 0.01).

## 4. Discussion

Matrix metalloproteinases (MMPs) are a family of zinc-dependent proteolytic enzymes essential for the degradation of extracellular matrix components. By this process, MMPs facilitate cellular migration, invasion, and signaling, making them indispensable for both normal cellular functions and the development of various diseases.

Among the MMPs, MMP-2 and MMP-9 are found in endothelial cells, vascular smooth muscle cells (VSMC), and adventitia [3]. MMP-2 is also expressed in dermal fibroblasts, leukocytes, and platelets, while MMP-9 is found in macrophages [3]. MMP-7, also known as matrilysin, is present in endothelial cells, VSMC, and the vascular wall, with similar substrates [3]. These MMPs are extensively studied for their roles in various pathologies, including atherosclerosis.

### 4.1. Influence of Statins on MMPs

A meta-analysis of randomized controlled trials on statin treatment concluded that, while statins effectively reduce the risk of cardiovascular disease, it remains unclear whether this benefit is primarily due to decreased LDLC levels or other mechanisms of action not yet fully understood. One limitation identified by this meta-analysis is the focus of most trials on participants with multiple heart disease risk factors, leaving the effect of statins in individuals without underlying clinical conditions underexplored [6]. This presents a significant ethical challenge, as it is problematic to administer medication to study participants who do not need it, despite statins being generally considered safe and offering multiple benefits beyond lipid-lowering effects. For instance, a retrospective cohort study on individuals without cardiovascular disease or major comorbidities found that short-term statin use (less than one year) was associated with an increased risk of long-term diabetes and diabetic complications without cardiovascular benefits [7]. Conversely, a large prospective study reported that rosuvastatin treatment significantly reduced the incidence of major cardiovascular events in healthy individuals with normal LDLC levels but elevated hsCRP, a cardiovascular risk factor [8].

Due to ethical challenges, few studies on the effects of statins have been conducted on healthy individuals. However, experimental models provide valuable data on the impact of statins. For example, statins inhibit the secretion of MMPs 1, 2, and 3 from VSMCs and macrophages [5]. Additionally, statins reduce the secretion of MMP-9 from macrophages [5,9,10], and rosuvastatin specifically decreases MMP-7 secretion by human monocyte-derived macrophages [11]. Statins also inhibit MMP-9 in human endothelial cells, potentially contributing to plaque stability [12]. In a rat model of heart failure, pravastatin suppressed the increase in myocardial MMP-2 and MMP-9 activity [13]. Similarly, simvastatin was effective in reducing the degree of myocarditis by inhibiting MMP activation in an experimental rat model [14].

Experimental findings indicate that statins exert regulatory effects on MMP expression. Consequently, numerous clinical studies have translated these findings in various contexts, yielding pertinent insights. For instance, long-term rosuvastatin therapy as part of broader treatment for hypertensive patients has been shown to reduce MMP-1 and MMP-9 levels while increasing TIMP-1 and TIMP-4 in circulation [15]. In hypercholesterolemic patients with type 2 diabetes, elevated MMP-7 and MMP-8 concentrations were reduced by atorvastatin, along with their ratios to TIMP-1, through suppression of inflammatory mediators [16]. Statin therapy has also demonstrated efficacy in decreasing MMP-3 and MMP-9 levels in patients with abdominal aortic aneurysms [17], as well as MMP-2 and MMP-9 levels in individuals with arterial aneurysmal disease [18]. In occlusive atherosclerotic conditions, statins have been observed to lower metalloproteinase levels in patients with carotid occlusive disease [19]. Furthermore, pre-hospital statin administration in ST-elevation myocardial infarction (STEMI) patients has been associated with reduced levels of proinflammatory markers (IL-6, CRP, TNF-α) and MMP-9 during hospitalization [20], and a loading dose of atorvastatin administered before percutaneous coronary intervention has been shown to decrease plasma MMP-9 levels and myocardial dysfunction in STEMI patients [21]. Combined therapy with ezetimibe and rosuvastatin in coronary artery disease patients has similarly resulted in significant reductions in plasma MMP-9 levels, potentially mitigating plaque instability and cardiovascular inflammation [22]. However, some studies have reported no effect of statin treatment on MMP plasma levels [23,24]. Importantly, none of these studies were conducted on healthy individuals, underscoring ethical constraints in studying drug effects in this population. Therefore, our study, albeit with a limited cohort, provides valuable information: statins (specifically atorvastatin) do not seem to influence plasma MMP levels in healthy individuals. This finding provides a baseline for interpreting changes in more complex pathological clinical scenarios.

### 4.2. MMPs and Atherosclerosis

MMPs play a key role in all stages of atherosclerosis. They contribute to vascular inflammation, endothelial dysfunction, smooth muscle cell migration, vascular calcification, extracellular matrix degradation, and plaque activation and destabilization [2,3,4]. The gelatinases, MMP-2 and MMP-9, as well as matrilysin (MMP-7), have been the focus of extensive research in various cardiovascular diseases, largely because of their crucial involvement in the regulation of VSMC migration and proliferation [25,26]. All this evidence of the involvement of MMPs in atherosclerosis at the molecular level has been further translated into clinical findings. For instance, an imbalance between MMPs and TIMPs has been observed in unstable carotid plaques, which is mirrored in the plasma levels of these markers [27]. Patients with significant carotid stenosis undergoing carotid endarterectomy and experiencing spontaneous embolization show higher plasma MMP-9 levels compared to those without embolization [28]. Furthermore, higher plasma levels of MMP-7 are linked to carotid calcification [29]. In patients with acute coronary syndrome, elevated plasma levels of MMP-9 and TIMP-1 indicate ongoing plaque rupture and an increased risk of subsequent cardiovascular events [30]. Elevated plasma levels of MMP-2 and MMP-9 in coronary heart disease patients correlate with plaque instability and the severity of acute coronary syndrome, making them valuable for predicting these events and diagnosing chronic total occlusion of the coronary artery [31]. Additionally, circulating MMP-2 and MMP-9 levels can reflect the effectiveness of treatment in heart failure patients and help identify those who may benefit from therapies targeting the MMP pathway [32].

However, in light of the study’s findings, this section will focus on the relationship between MMP-7 and atherosclerosis. Several studies suggest the involvement of MMP-7 in atherosclerosis. At the genetic level, functional polymorphisms in the *MMP7* gene have been linked to cardiovascular disease susceptibility and outcomes [33]. Additionally, an association between coronary artery luminal dimension and allele-specific regulation of MMP-7 promoter activity has been observed in hypercholesterolemic individuals [34]. Clinically, MMP-7 has been associated with cardiovascular diseases in various contexts, including carotid atherosclerosis [35], chronic kidney disease [36], and type 2 diabetes with hypercholesterolemia [16].

In this study, pre-treatment MMP-7 was positively correlated with age and most atherogenic lipid profile parameters, including TC, dLDLC, non-HDLC, remnant cholesterol, TG, TC/HDLC, dLDLC/HDLC, and AIP, but not HDLC. Similarly, pre-treatment hsCRP showed positive correlations with the same lipid parameters, but it was significantly and negatively correlated with HDLC. Additionally, pre-treatment MMP-7 was positively correlated with white blood cell count, neutrophils, and neutrophil-derived parameters such as NLR, dNLR, and NHR, while pre-treatment hsCRP showed no such correlation with these neutrophil-related inflammatory markers. The role of dyslipidemia in atherosclerosis is well established, while the involvement of neutrophils has been recently elucidated, these cells being actively involved in the process of atherosclerosis and higher neutrophil counts being linked to increased atherosclerosis-related events [37,38].

Interestingly, despite no significant effect of statin treatment on plasma MMP levels, the correlation between MMP-7 and both lipid and neutrophil-related biomarkers disappeared after one month of moderate-dose atorvastatin treatment. While this might initially be attributed to the lipid-lowering effects of atorvastatin, from a mathematical perspective, a proportional reduction in lipid levels should not alter statistical correlations if MMP levels remain unchanged. This suggests that atorvastatin’s effects are more complex, potentially disrupting the relationship between MMP-7 and these biomarkers.

Given the anti-inflammatory effects of statins [39,40], the elevated levels of MMPs in inflammatory states [41,42], and the observation that atorvastatin in this study slightly but significantly reduced hsCRP, a pertinent question arises: Did atorvastatin disrupt the pre-treatment association of MMP-7 with blood lipids and neutrophil-related inflammatory markers, via a reduction in inflammation? This does not appear to be the case, at least not in a conventional sense, and within the methodological frame of the present study. First, while both pre-treatment MMP-7 and hsCRP were similarly associated with blood lipids, they were not correlated with each other, and only MMP-7 was correlated with neutrophil-related inflammatory parameters. Second, post treatment, hsCRP remained correlated with blood lipids and maintained its lack of correlation with MMP-7, while MMP-7 lost its correlation with both blood lipids and neutrophil-related parameters. Taken together, these findings suggest that atorvastatin’s lipid-lowering effects are more complex, potentially disrupting the relationship between MMP-7 and blood lipids and neutrophil-related inflammatory markers independently of mainstream inflammatory pathways, as indicated by CRP, a general marker of inflammation. This disruption may be attributed to the pleiotropic effects of statins, which extend beyond lipid reduction by influencing various metabolic pathways. Consequently, even in healthy individuals, statins might modulate the interplay between MMP-7 and biomarkers related to atherosclerosis. Given the small cohort size in this study, further research is necessary to validate these findings and fully understand the mechanisms involved.

## 5. Conclusions

MMPs have been extensively studied in relation to cardiovascular disease. Experimental studies have consistently shown that statins reduce MMP levels at the cellular and tissue levels. However, data on how statin treatment affects plasma levels of MMPs are contradictory. Statins seem to reduce plasma levels of MMPs in patients with cardiovascular and related diseases, but no information is available for healthy individuals without cardiovascular comorbidities or current medication.

In our small group of healthy participants, we found that short-term, moderate-dose atorvastatin treatment did not influence plasma levels of MMPs 2, 7, and 9. While no significant correlations were found for MMPs 2 and 9, MMP-7 showed a significant positive correlation with age and known atherosclerosis risk factors, such as total cholesterol, low-density lipoprotein cholesterol, and blood count-derived neutrophil-associated inflammatory markers. However, this correlation disappeared after statin treatment independently of C-reactive protein, suggesting that atorvastatin might modulate the relationship between MMP-7 and plasma lipids through mechanisms beyond its lipid-lowering or anti-inflammatory effects, possibly via pleiotropic effects.

## Figures and Tables

**Table 1:** General data and laboratory parameters at baseline and after statin treatment.

	General Data		
	Median	Range	IQR
Sex	n = 21 (8 male, 13 female)		
Age (years)	28.00	25.00–63.00	26.00–49.25
BMI (kg/m^2^)	23.60	19.50–33.10	21.20–24.47
Treatment duration (days)	28.00	26.00–31.00	27.75–28.00
	Chemistry tests		
	Baseline	After treatment	*p*-value
Albumin (g/dL)	4.63 [4.47–4.73]	4.66 [4.52–4.77]	-
AST (U/L)	26.4 [21.1–29.1]	29.2 [24.9–31.3]	0.035
ALT (U/L)	11.4 [9.3–16.8]	13.8 [10.2–23.8]	0.002
AST/ALT	1.79 [1.61–2.50]	1.85 [1.45–2.57]	-
Amylase (U/L)	65.0 [51.0–80.4]	70.7 [53.9–80.3]	-
CK (U/L)	92.2 [71.0–112.5]	100.4 [75.5–136.2]	0.022
Creatinine (mg/dL)	0.72 [0.64–0.86]	0.73 [0.66–0.81]	-
GGT (U/L)	21.6 [16.1–30.1]	23.4 [18.3–38.7]	-
Glucose (mg/dL)	84.0 [78.0–89.0]	86.0 [76.2–91.7]	-
LDH (U/L) ^1^	371.2 [240.9–526.2]	446.0 [384.4–535.8]	0.049
hsCRP (mg/L) ^2^	0.70 [0.40–2.27]	0.60 [0.30–1.10]	0.025
	Blood count		
WBC (×10^3^/µL)	6.04 [5.55–7.00]	6.54 [5.06–7.71]	-
Neutrophil (×10^3^/µL)	3.21 [2.80–3.66]	3.46 [2.54–4.42]	-
Lymphocyte (×10^3^/µL)	2.12 [1.89–2.43]	2.26 [1.72–2.65]	-
Monocyte (×10^3^/µL)	0.48 [0.39–0.58]	0.51 [0.40–0.60]	-
MLR	0.23 [0.17–0.27]	0.22 [0.17–0.28]	-
NLR	1.51 [1.16–1.98]	1.63 [1.26–2.33]	-
dNLR	1.09 [0.93–1.50]	1.27 [0.97–1.64]	-
SIRI	0.75 [0.55–0.96]	0.85 [0.53–1.11]	-
NHR	62.2 [51.2–89.3]	73.5 [52.1–85.9]	-
MHR	9.6 [7.0–12.5]	9.4 [6.5–14.0]	-
LHR	41.1 [33.6–55.8]	39.7 [29.0–55.6]	-
	Lipid profile		
	Baseline	After treatment	*p*-value
TC (mg/dL)	194.8 [166.3–222.9]	134.0 [112.0–142.5]	0.0001
HDLC (mg/dL)	52.7 [37.8–65.0]	53.3 [41.5–60.9]	-
dLDLC direct (mg/dL)	116.3 [87.1–155.6]	66.3 [51.9–77.5]	0.0001
LDLC calculated (mg/dL)	110.4 [82.5–143.6]	61.8 [48.7–73.6]	0.0001
Non-HDLC (mg/dL)	149.0 [100.1–174.1]	76.8 [57.6–98.5]	0.0001
TG (mg/dL)	81.1 [70.4–117.0]	66.6 [51.4–96.8]	0.001
Lipoprotein(a) (mg/dL) ^3^	14.6 [12.5–24.4]	16.4 [16.1–23.2]	-
TC/HDLC	3.67 [2.78–4.50]	2.47 [2.10–2.94]	<0.0001
dLDLC/HDLC	2.51 [1.72–3.08]	1.30 [0.94–1.64]	<0.0001
AIP	0.17 [0.03–0.40]	0.06 [−0.02 to 0.21]	0.0021
	MMPs		
	Baseline	After statins	*p*-value
MMP-2 (ng/mL)	136.3 [120.2–164.0]	147.8 [134.3–163.2]	-
MMP-7 (ng/mL)	17.3 [12.4–23.6]	18.3 [11.6–27.2]	-
MMP-9 (ng/mL)	64.8 [46.8–80.5]	84.9 [54.1–103.3]	-

Only *p*-values ≤ 0.10 were reported. Data are reported as median with [IQR]. Abbreviations: AIP—atherogenic index of plasma, ALT—alanine transaminase, AST—aspartate aminotransferase, BMI—body mass index, CK—creatine kinase, dNLR—derived NLR, GGT—gamma-glutamyl transferase, HDLC—high-density lipoprotein cholesterol, IQR—interquartile range, LDH—lactate dehydrogenase, dLDLC—low-density lipoprotein cholesterol (measured), LHR—lymphocyte-to-HDLC ratio, MHR—monocyte-to-HDLC ratio, MLR—monocyte-to-lymphocyte ratio, MMP—matrix metalloproteinase, NHR—neutrophil-to-HDLC ratio, NLR—neutrophil-to-lymphocyte ratio, SIRI—systemic inflammation immune index, TC—total cholesterol, TG—triglyceride, WBC—white blood cell. ^1^ LDH values are known to be elevated in plasma compared to serum. ^2^ Analysis was performed on 19 participants after excluding two individuals with hsCRP levels indicative of acute inflammation (one with 41 mg/L before treatment and one with 14.5 mg/L after treatment). ^3^ Analysis was performed on 5 participants. The other 16 individuals had lipoprotein(a) levels below the limit of quantification (<8.5 mg/dL) both before and after treatment.

**Table 2:** Correlation between MMP levels and other parameters.

	MMP-2		MMP-7		MMP-9	
	Before	After	Before	After	Before	After
MMP-2	-	-	0.29	0.58 ***	−0.35	0.54 **
MMP-7	0.29	0.58 ***	-	-	−0.05	0.39 ^#^
MMP-9	−0.35	0.54 **	−0.05	0.39 ^#^	-	-
Age	−0.13	−0.29	0.51 *	−0.11	−0.14	−0.37 ^#^
BMI	−0.52 *	0.34	0.11	0.46 *	−0.02	0.47 *
hsCRP	−0.02	−0.03	0.20	0.26	0.00	0.08
TC	0.17	0.10	0.50 *	0.03	−0.26	−0.44 *
dLDLC	0.11	0.09	0.45 *	0.13	−0.34	−0.41 ^#^
HDLC	0.23	0.26	−0.27	−0.09	−0.27	−0.17
nHDLC	0.10	0.04	0.56 **	0.16	−0.20	−0.38 ^#^
RemnantC	−0.06	−0.15	0.49 *	0.08	0.37 ^#^	−0.06
TG	0.02	−0.16	0.50 *	−0.01	0.33	−0.28
TC/HDLC	−0.05	−0.18	0.60 ***	−0.02	0.02	−0.25
LDLC/HDLC	0.01	−0.17	0.59 ***	0.20	−0.03	−0.27
AIP	−0.10	−0.33	0.45 *	0.04	0.36	−0.15
WBC	−0.18	−0.14	0.40 ^#^	0.28	0.30	0.16
NEU	−0.15	−0.39 ^#^	0.44 *	0.11	0.31	0.19
NLR	−0.02	−0.40 ^#^	0.37 ^#^	−0.31	0.12	0.37 ^#^
dNLR	−0.03	−0.40 ^#^	0.37 ^#^	−0.27	0.14	0.41 ^#^
NHR	−0.20	−0.41 ^#^	0.48 *	0.12	0.30	0.17
LY	−0.16	0.23	0.12	0.48 *	0.26	0.00
LHR	−0.27	−0.17	0.26	0.45 *	0.35	0.15
MONO	−0.28	−0.15	0.00	0.06	0.05	0.28
MLR	−0.09	−0.33	−0.17	−0.50 *	−0.16	0.27
MHR	−0.31	−0.36	0.15	0.08	0.31	0.30
SIRI	−0.10	−0.48 *	0.30	−0.21	0.12	0.35

Data are reported as correlation coefficients, either Pearson’s r or Spearman’s ρ, depending on the characteristics of each dataset. Significance thresholds: ^#^ *p* ≤ 0.10, * *p* ≤ 0.05, ** *p* ≤ 0.01, *** *p* ≤ 0.005. Abbreviations: AIP—atherogenic index of plasma, BMI—body mass index, dNLR—derived NLR, HDLC—high-density lipoprotein cholesterol, dLDLC—low-density lipoprotein cholesterol (measured), LHR—lymphocyte-to-HDLC ratio, MHR—monocyte-to-HDLC ratio, MLR—monocyte-to-lymphocyte ratio, MMP—matrix metalloproteinase, NHR—neutrophil-to-HDLC ratio, NLR—neutrophil-to-lymphocyte ratio, SIRI—systemic inflammation immune index, TC—total cholesterol, TG—triglyceride, WBC—white blood cell.

**Table 3:** Simple linear regression models for calculation of pre-treatment plasma MMP-7 levels.

	Intercept [95% CI]	Slope [95% CI]	R^2^	*p*-Value
TC/HDLC	4.43 [−4.10–12.97]	3.338 [1.229–5.447]	0.36	0.003
dLDLC/HDLC	6.97 [−1.15–15.09]	4.176 [1.087–7.264]	0.29	0.010
nHDLC	5.47 [−3.71–14.67]	0.084 [0.021–0.146]	0.29	0.011
Age	7.32 [−1.11–15.74]	0.269 [0.055–0.483]	0.26	0.016
TG	12.90 [8.31–17.49]	0.037 [0.006–0.068]	0.25	0.020
RemnantC	14.41 [10.69–18.12]	0.182 [0.028–0.335]	0.24	0.022
AIP	14.83 [11.12–18.55]	9.096 [0.465–17.728]	0.20	0.039
TC	5.92 [−6.68–18.53]	0.058 [−0.004–0.121]	0.16	0.066
LDLC	9.08 [−1.05–19.22]	0.065 [−0.011–0.143]	0.14	0.090

Abbreviations: AIP—atherogenic index of plasma, CI—confidence interval, nHDLC—non-HDL cholesterol. RemnantC—remnant cholesterol, TC—total cholesterol, TG—triglyceride.

## Data Availability

The raw data supporting this study’s findings are openly available in FigShare at the following DOI: 10.6084/m9.figshare.26114503.
